# The aid of microsurgical instruments in nasolabial cyst enucleation. A report of two cases with critical review of the therapeutic approach

**DOI:** 10.1093/jscr/rjad011

**Published:** 2023-01-25

**Authors:** Ioannis Tilaveridis, Gregory Venetis, Dimitris Tatsis, Ioanna Kalaitsidou, Lambros Zouloumis

**Affiliations:** Department of Oral and Maxillofacial Surgery, Aristotle University of Thessaloniki, Thessaloniki, Greece; Department of Oral and Maxillofacial Surgery, Aristotle University of Thessaloniki, Thessaloniki, Greece; Department of Oral and Maxillofacial Surgery, Aristotle University of Thessaloniki, Thessaloniki, Greece; Oberärztin, Universitätsklinik für Schädel-, Kiefer- und Gesichtschirurgie, Inselspital, Universitätsspital Bern, Freiburgstrasse, Bern, Switzerland; Department of Oral and Maxillofacial Surgery, Aristotle University of Thessaloniki, Thessaloniki, Greece

## Abstract

Nasolabial cysts are rare non-odontogenic cysts related to epithelial remnants of the nasolacrimal duct, slowly enlarging and provoking extraoral swelling in the nasolabial fold with obstruction of the naris. Two patients of large unilateral nasolabial cysts are reported, appearing as cosmetically unappealing distention of the nasolabial fold. Diagnosis is based on clinical characteristics. Computed tomography imaging reveals the dimensions of the cyst, the correlation to the nasal cavity and might depict a depression on the labial surface of the maxilla. Intraoral surgical enucleation of the cystic wall or nasal marsupialization is the main treatment modalities. However, both procedures are related to a small percentage of recurrence. Microsurgical instruments were used to dissect the cystic wall from the nasal mucosa. The aim of this paper is to stress the implementation of microsurgical instruments to separate the cystic wall of the nasolabial cyst from the thin and friable nasal mucosa to avoid recurrence.

## INTRODUCTION

A nasolabial cyst is a benign cystic lesion affecting the soft tissue near the ala nasi, classified as a developmental non-odontogenic cyst. It develops purely into the soft tissues despite its classification in jaw cysts. The cyst’s nomenclature varied and was initially described as Klepstadt’s cyst in 1882, until 1952 when Rao described this cyst as nasolabial, the current term [1–4].

Nasolabial cysts typically appear in the fourth and fifth decade of life and affects females to males in a ratio of 4:1. It is a rare lesion affecting 1.6 per 100.000 of the population and comprises 0.1–0.6% of the reported jaw cysts [2, 3].

This paper aims to stress the importance of microsurgical instruments to separate the cystic wall of the nasolabial cyst from the thin and friable nasal mucosa to avoid recurrence and postoperative complications. Even though the cystic wall is thick enough to dissect it from the surrounding soft tissue, sometimes the dissection and the separation from the nasal mucosa is difficult and can result in tearing with the possibility of fistula formation. For this reason, the use of microsurgical instruments is proposed to dissect the cystic wall from the nasal mucosa.

## CASE REPORT

### Case presentation 1

**Figure 1:**
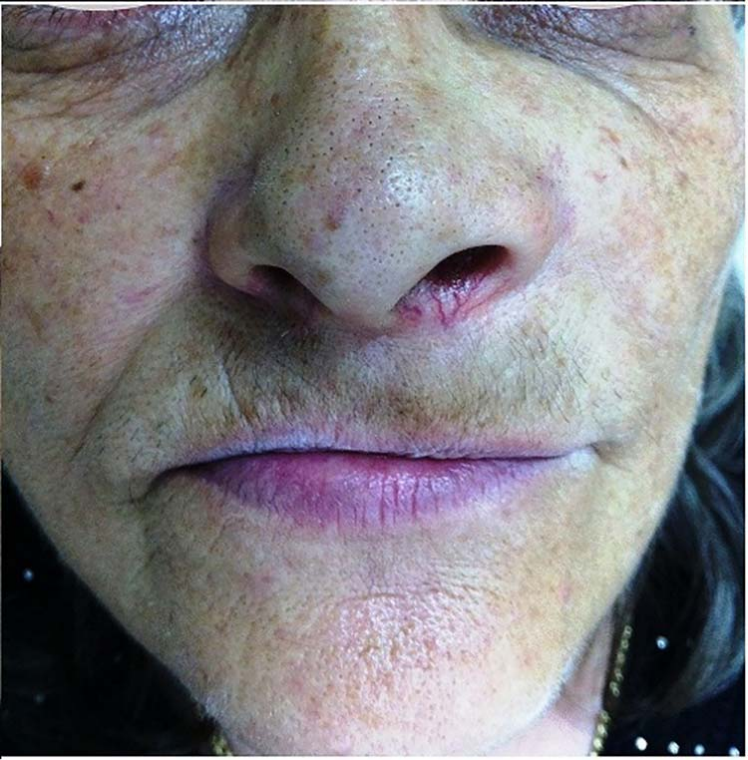
Pre-operative clinical presentation of patient (case 1).

**Figure 2:**
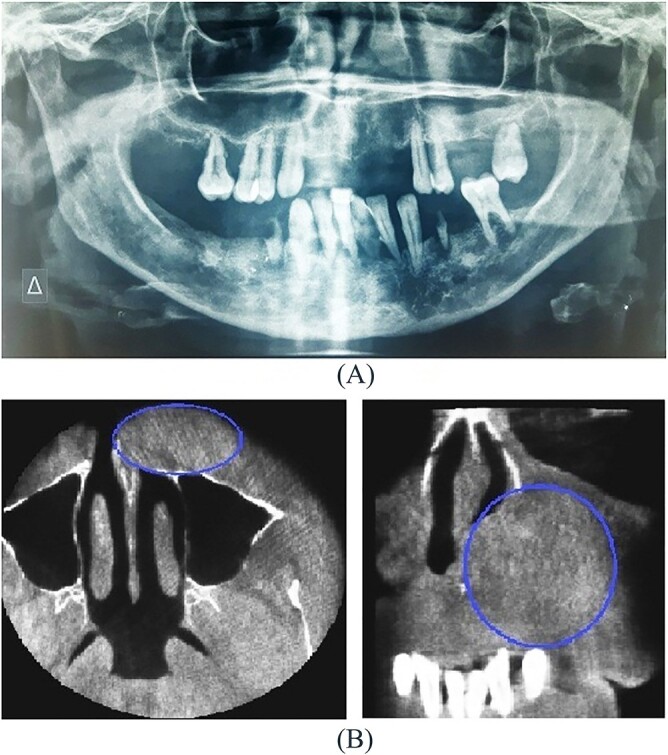
(**A**) Orthopantomogram, (**B**) CBCT imaging of soft tissue mass.

A 54-year-old-Caucasian female was referred to our department with a swelling of the left maxillary area (Fig. 1), actively inflaming. Antibiotics were prescribed, and after a week, the swelling persisted but was painless, roundish, fluctuant in palpation, sizing ~3 cm in diameter. The overlying mucosa was normal and mobile over the lesion. Clinical examination also revealed an extraoral asymmetry over the nasolabial sulcus because of the presence of this lesion causing obstruction of the left nostril. The patient presented with poor oral hygiene and the orthopantomogram revealed periodontal disease (Fig. 2a). CBCT imaging revealed a low density, ovoid, cystic soft tissue mass of 3.4 × 2.3 × 3 cm in close contact with the alar base. The maxilla near the lesion appeared concave, because of the cyst’s pressure, but without erosion (Fig. 2b). The patient was advised to undergo a contrast-enhanced computed tomography (CT) scan, but she did not wish to perform any other imaging study. The patient’s medical history was non-contributory.

**Figure 3:**
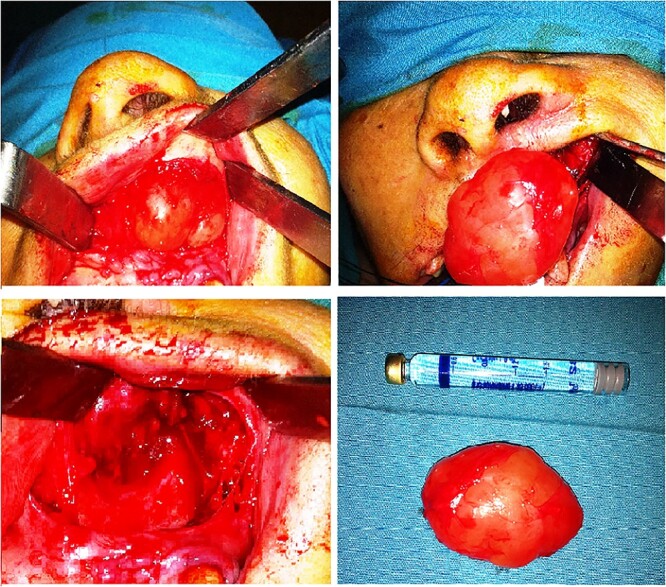
Surgical approach- enucleation, surgical specimen.

Under general anesthesia, an intraoral incision at the upper left gingival buccal sulcus and a meticulous dissection of the cystic wall was performed. The cystic wall was thick without presenting any sign of rupture. However, near the nose, loupes magnification (5×) and microsurgical instruments were utilized to dissect the cystic wall from the thin and friable nasal mucosa. The cyst was enucleated completely without any tearing of the cystic wall (Fig. 3). The overlying nasal mucosa also remained intact and after wound inspection, the wound closed with interrupted sutures with polyglactin 4/0 sutures.

**Figure 4:**
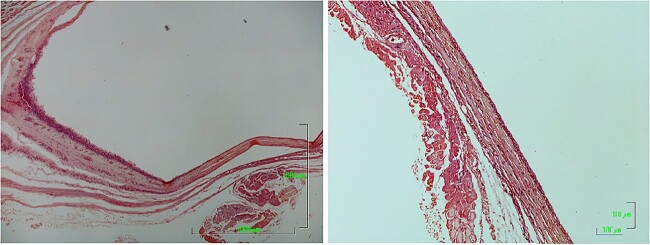
Histological section of cyst [Hematoxylin—Eosin].

The histologic examination of the surgical specimen revealed a cystic wall covered with pseudostratified columnar epithelium with basaloid and few goblet cells. Cellular atypia was not detected in any area of the specimen, and the final diagnosis was a nasolabial cyst (Fig. 4).

The postoperative course was uneventful and the extraoral swelling fully subsided 3 months after the operation. Two years later, no sign of recurrence was evident.

### Case presentation 2

**Figure 5:**
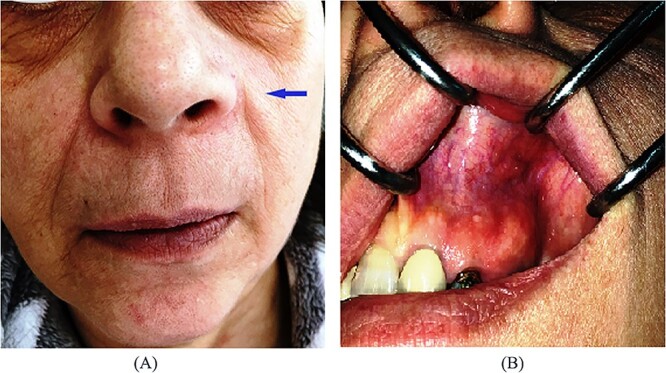
(**A**) Extraoral, (**B**) intraoral pre-operative clinical presentation of patient (case 2).

**Figure 6:**
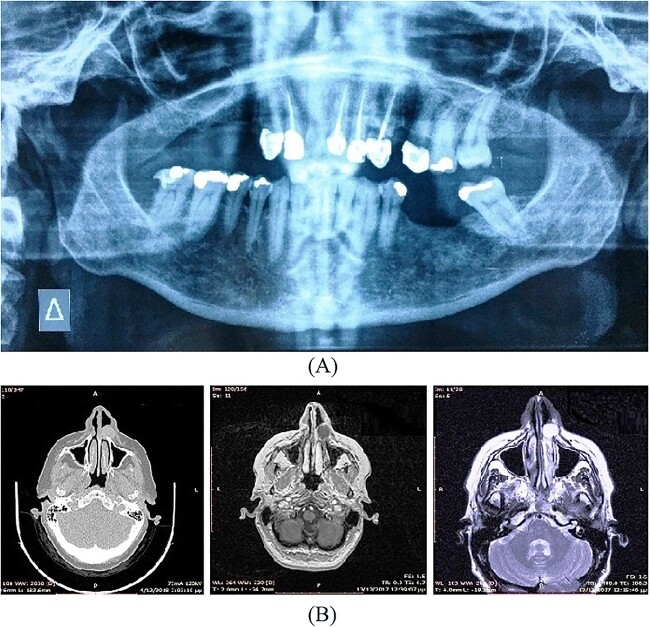
(**A**) Orthopantomogram (**B**) CT, MRI image of the lesion.

A 57-year-old-Caucasian female patient was referred to our department, complaining of swelling of the left anterior maxillary area (Fig. 5a). The patient had been aware of the swelling for 20 months, but without any pain or tenderness. Physical examination revealed a 2-cm soft lump with normal overlying mucosa and mobile over the lesion (Fig. 5b). An extraoral asymmetry over the left nasolabial sulcus and obstruction of the left nostril was observed. The teeth, adjacent to the lesion, were endodontically treated. The cyst was not identified on the panoramic radiograph of the jaws (Fig. 6a). CT and magnetic resonance imaging (MRI) images showed an oval lesion with slight peripheral enhancement emanating from the lateral wall of the left nasal cavity (Fig. 6b). The lesion, measured 1.7 × 1.9 × 1.3 cm, was considered a simple cystic lesion. No erosion was evident on the underlying maxillary bone, despite its extension anteriorly to it.

**Figure 7:**
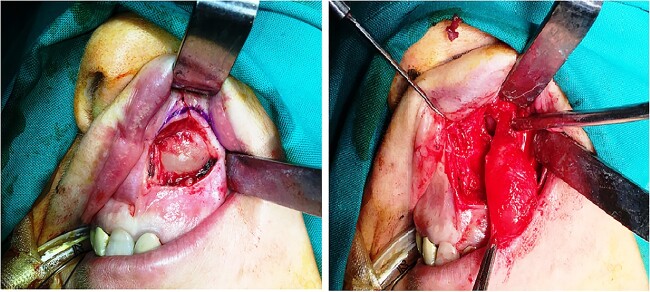
Surgical approach-enucleation.

Under general anesthesia, complete surgical excision of the cyst was performed via an intraoral incision at the upper left gingival buccal sulcus. As in the previous case, loupes magnification (5×) and microsurgical instruments were utilized near the nose to dissect the cystic wall from the thin and friable nasal mucosa. The cyst was enucleated completely without any tearing of the cystic wall (Fig. 7). The overlying nasal mucosa also remained intact and after wound inspection, the wound closed with interrupted polyglactin 4/0 sutures.

**Figure 8:**
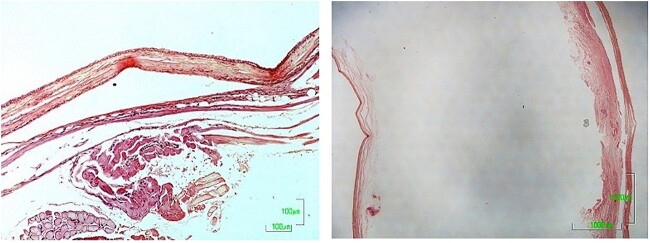
Histological section of cyst [Hematoxylin—Eosin].

Histopathological examination revealed a cystic lesion, mostly lined with a thin layer of stratified squamous epithelium, with a fibrous capsule and chronic inflammatory changes of varying intensity, consistent with a nasolabial cyst (Fig. 8).

Postoperative period was uneventful. A clinical and radiological follow-up examination yielded no recurrence almost 1 year after the surgery.

## DISCUSSION

Nasolabial cysts are benign non-odontogenic lesions that arise in the anterior maxillary region. The pathogenesis of the cyst is well described in the literature. It is hypothesized that it originates from epithelial remnants that remain during the fusion of lateral nasal and median processes [5]. According to other investigators, it comes from the peripheral part of the nasolacrimal duct epithelium [5, 6].

Nasolabial cysts have the highest prevalence in female adults between 40 and 50 years old [1, 7]. Clinical presentation of the lesion usually includes a painless swelling in the nasolabial area. The swelling may result in extraoral asymmetry of the face and may project intraorally [1, 2, 8]. The course of the lesion is a slowly enlarged mass that may develop ipsilateral nasal obstruction. The mass is soft, fluctuant and painless in palpation except for cases with secondary infection [5, 8]. A well-located fluctuating swelling with a cystic consistency in the nasolabial sulcus is a definitive sign of a nasolabial cyst [9]. Schuman reported that 65% of the patients had symptoms for over 12 months before a diagnosis was made [10]. Both of the presented patients were females, 54 and 57 years old, respectively. The first patient was referred for the painful swelling of the right nasolabial region. However, the main complaint of the second patient was cosmetically unappealing distention of the nasolabial fold due to a swelling that also incorporated and raised the lateral nasal ala.

Imaging includes an orthopantomogram to exclude other odontogenic lesions, and CT imaging to estimate the exact dimensions and the extension of the cyst to the neighboring structures of the nose. Imaging typically records a homogeneous, non-contrast enhancing cystic lesion anterior to the piriform opening. Remodeling of the underlying maxillary bone may be seen in larger cysts as well. Both present cases demonstrated in imaging well-defined cystic lesions in deep lateral nasal areas. On MRI, these cysts can appear as homogeneous intermediate intensity T1 signals and homogeneous high-intensity T2 signals. However, CT imaging is the preferred option in the pre-operative diagnosis of nasolabial cysts [11].

Diagnosis is based on clinical examination and aided by CT or MRI imaging and confirmed by histologic examination [1, 8]. Histopathology of the cystic wall reveals a ciliated pseudostratified columnar epithelium and occasionally a stratified squamous epithelium lining the cystic lumen. Scattered interspersed luminal goblet cells are frequently also observed in those cases. Focal squamous metaplasia or apocrine changes may also be present. The histologic features of nasolabial cysts show a striking similarity to the physiologically encountered epithelia in the lacrimal drainage system, specifically the lacrimal canaliculi and sac and the nasolacrimal duct [5]. Histopathology was performed in both excised surgical specimens.

The differential diagnosis includes odontogenic and non-odontogenic cysts or granuloma of the maxilla and cysts of the soft tissue [1, 2]. Such entities are: Radicular cyst with soft tissue expansion, periapical cyst, dentigerous cyst and rarel, nasopalatine duct cyst. Benign soft tissue neoplasms such as odontogenic keratocyst tumor, schwannomas, neurofibromas, minor salivary gland tumors, heterotopic gastrointestinal cyst, dermoid or epidermoid cyst should also be considered in the differential diagnosis [12, 13]. The long-term pressure phenomena of a cyst may result in resorption of the underlying maxilla, thereby demanding the differential diagnosis from further osseous or extraosseous lesions [1, 3, 8].

Treatment of nasolabial cyst is the surgical enucleation including the whole cystic wall, marsupialization through the nose with endoscopic assistance, or the use of sclerosing or intralesional injections [13]. However, both intraoral resection and endoscopic trans nasal marsupialization are associated with similar possibilities of recurrence after surgical treatment [12, 13]. In the present cases, the intraoral enucleation technique was used with a sublabial approach followed by dissection along the surgical planes to the piriform aperture. Microsurgical instruments were used to dissect the cystic wall from the thin and friable nasal mucosa. The microdissection of the cystic wall through the intraoral approach offers a better approach to minimize postoperative complications, such as recurrence or oronasal communication.

In conclusion, nasolabial cysts are exceedingly rare benign extraosseous soft tissue lesions, 0.1% of all jaw cysts [13]. Morphological studies support the hypothesis of developmental disorders of the nasolacrimal duct as the cells of origin giving rise to nasolabial cysts. No cases of malignant transformation have been reported to our knowledge. Careful and complete surgical resection provides a definitive cure with minimal recurrence rates.

## CONFLICT OF INTEREST STATEMENT

The authors report no conflicts of interest related to this study.

## FUNDING

None.
